# Genetically encoded ratiometric fluorescent thermometer with wide range and rapid response

**DOI:** 10.1371/journal.pone.0172344

**Published:** 2017-02-17

**Authors:** Masahiro Nakano, Yoshiyuki Arai, Ippei Kotera, Kohki Okabe, Yasuhiro Kamei, Takeharu Nagai

**Affiliations:** 1 The Institute of Scientific and Industrial Research, Osaka University, Ibaraki, Osaka, Japan; 2 Research Institute for Electronic Science, Hokkaido University, Sapporo, Hokkaido, Japan; 3 Graduate School of Pharmaceutical Sciences, The University of Tokyo, Bunkyo-ku, Tokyo, Japan; 4 JST, PRESTO, Kawaguchi, Saitama, Japan; 5 National Institute for Basic Biology, Okazaki, Aichi, Japan; Okayama Daigaku, JAPAN

## Abstract

Temperature is a fundamental physical parameter that plays an important role in biological reactions and events. Although thermometers developed previously have been used to investigate several important phenomena, such as heterogeneous temperature distribution in a single living cell and heat generation in mitochondria, the development of a thermometer with a sensitivity over a wide temperature range and rapid response is still desired to quantify temperature change in not only homeotherms but also poikilotherms from the cellular level to *in vivo*. To overcome the weaknesses of the conventional thermometers, such as a limitation of applicable species and a low temporal resolution, owing to the narrow temperature range of sensitivity and the thermometry method, respectively, we developed a genetically encoded ratiometric fluorescent temperature indicator, gTEMP, by using two fluorescent proteins with different temperature sensitivities. Our thermometric method enabled a fast tracking of the temperature change with a time resolution of 50 ms. We used this method to observe the spatiotemporal temperature change between the cytoplasm and nucleus in cells, and quantified thermogenesis from the mitochondria matrix in a single living cell after stimulation with carbonyl cyanide 4-(trifluoromethoxy)phenylhydrazone, which was an uncoupler of oxidative phosphorylation. Moreover, exploiting the wide temperature range of sensitivity from 5°C to 50°C of gTEMP, we monitored the temperature in a living medaka embryo for 15 hours and showed the feasibility of *in vivo* thermometry in various living species.

## Introduction

Temperature is a fundamental parameter in biological reactions and events because it affects the diffusion of biomolecules, enzymatic activity, heat shock-induced gene expression [1], and the sex determination of poikilotherms such as reptiles [2] and fish [3]. However, the spatiotemporal patterns of temperature at the single cell level remain largely unknown. To investigate this, several indicators have been developed to date [4–13]. Although previously developed thermometers have enabled new findings, such as heterogeneous temperature distribution in a single living cell [6, 11, 13] and heat generation in mitochondria [4, 6, 8, 13], a thermometer exhibiting a wide range and rapid response is required to quantify the temperature change in not only homeotherms, such as mammals, but also poikilotherms from the cellular level to *in vivo*. For this purpose, fluorescent protein (FP)-based thermometers are essential for noninvasive temperature imaging in living things. Previously, enhanced green fluorescent protein (EGFP) was used for temperature measurements of the micro environment in the range from 20°C to 60°C, and showed a rapid response to irradiation with an infrared (IR) laser [14]. This thermometry method, based on a fluorescence intensity change of a single FP, is simple and enables fast (on the order of milliseconds) measurement but is influenced by other factors such as the expression level of the FP and the shape of the specimen. To overcome these drawbacks, some FP-based thermometry methods have been developed. For example, Donner *et al*. developed a temperature monitoring method using GFP by measuring the fluorescence polarization anisotropy, which depends on temperature [7]. This method enabled the quantitative measurement of the temperature in cells with an accuracy of approximately 0.4°C using confocal microscopy. However, their thermometry method required 20 ms per pixel, indicating that it would take several minutes for imaging a whole cell. Such a slow temporal resolution would also make it difficult to track a fast temperature change inside cells. Kiyonaka *et al*. developed fluorescence intensity ratiometric thermometers, tsGFPs, which possess a thermosensing protein, TlpA, and wild-type GFP [8]. However, tsGFPs do not exhibit temperature sensitivity below 35°C, making it difficult to measure the temperature in poikilotherms. Moreover, tsGFPs require two different wavelengths of excitation light to enable temperature imaging. Therefore, tsGFPs are not suitable for monitoring fast temperature changes. For these reasons, we have developed a fluorescent thermometer applicable to various organisms *in vivo* and having the ability to track biological events with fast temperature change.

## Materials and methods

### Gene construction

To introduce the A206K mutation into cDNAs of T-Sapphire, we performed Quik change reactions for site-directed mutagenesis. Sirius, mTurquoise2, mTFP1, EGFP, Venus, TagRFP-T, and mCherry were already subcloned into a pRSET_B_ (Invitrogen) vector in our laboratory. cDNA of mT-Sapphire was subcloned into a pRSET_B_ vector with 5’ *Bam*HI and 3’ *EcoR*I restriction enzyme (RE) sites. To construct the tandem Sirius-mT-Sapphire fusion, Sirius cDNA was amplified using PCR to contain 5’ *Bam*HI and 3’ *Xho*I RE sites. The cDNA of mT-Sapphire was also amplified using PCR to contain 5’ *Xho*I and 3’ *Eco*RI RE sites. Then, the fragments were digested, ligated and subcloned into *Bam*HI/*Eco*RI sites of a pRSET_B_ vector. For mammalian expression, the cDNAs of Sirius were amplified using PCR to contain 5’ *Bam*HI and 3’ *Sph*I RE sites and the cDNAs of mT-Sapphire were amplified using PCR to contain 5’ *Sac*I and 3’ *Eco*RI sites. The T2A peptide with a GSG amino-acid linker containing *Sph*I and *Sac*I RE sites was made by annealing forward and reverse primers. The *Bam*HI/*Sph*I fragments, *Sph*I/*Sac*I fragments, and *Sac*I/*Eco*RI fragments were ligated and inserted into *Bam*HI/*Eco*RI sites of a pcDNA3 (Invitrogen) vector. A duplex of the mitochondrial targeting signal of cytochrome *c* oxidase subunit VIII was fused to each N-terminus of Sirius and mT-Sapphire in the pcDNA3 vector for mitochondrial targeting. Histone 2B was fused to each C-terminus of Sirius and mT-Sapphire in the pcDNA3 vector for nucleus targeting.

### Protein purification

*E*. *coli* (JM109(DE3)) was transformed with pRSET_B_ vectors encoding a FP gene and grown for 60 hours at 23°C with gentle shaking at 150 rpm. FPs were purified until buffer exchanging (20 mM HEPES, pH 7.4) following the methods of a previous report [15].

### Measurement of fluorescence spectra *in vitro*

The fluorescence spectra of purified FPs were measured using a FP-750 spectrofluorometer (JASCO) equipped with a temperature controller unit (ETC-272T, JASCO). The wavelength at 360 ± 10 nm was used for excitation. 20 mM MOPS-NaOH (pH 7.3) with 150 mM NaCl was used for assays.

### Culture and transfection for HeLa cells

HeLa cells were purchased from RIKEN BioResource Center and grown in Dulbecco’s modified Eagle’s medium (DMEM) containing 10% heat-inactivated fetal bovine serum (FBS) at 37°C in a 5% CO_2_ incubator. One day before transfection, cells were dissociated and transferred onto a custom-made glass bottom dish with a 35-mm cell culture dish (430165, NUNK) and a coverglass (No.1S grade, Matsunami Glass)). 4.0 μg of plasmid DNA was mixed with calcium phosphate buffer (25 mM HEPES-NaOH, pH 7.1, 140 mM NaCl, 0.7 mM Na_2_HPO_4_ and 125 mM CaCl_2_) and then we added the mixture to the cell. After 10–12 hours, the cells were washed with DMEM containing 10% FBS and cultured for 20–40 hours.

### Making HeLa cell line stably express gTEMP

After transfecting the gTEMP gene to HeLa cells, the cells were cultured with DMEM supplemented with 10% FBS and 600 μg/ml geneticin (G418) until a colony of the cells formed. The fluorescent colonies were isolated and cultured with the same medium to increase the cell number.

### Cellular imaging by microscopy

For imaging of gTEMP, cells were visualized with an inverted microscope (Ti-E, Nikon) equipped with a 60× 1.4 numerical aperture oil immersion objective lens (PlanApo λS, Nikon), motorized-stage (BIXY Chuo Precision Industrial Corp.), and stage-top incubator (INUB-ONICS, Tokai Hit). Cells were illuminated using Intensilight (Nikon) through 12.5% and 25% neutral density filters. The excitation filter FF01-370/36 (Semrock), CFW-Di01-Clin dichroic mirror (Semrock), and emission filters (FF01-447/60 and FF01-520/35 for Sirius and mT-Sapphire, respectively; Semrock) were used with a filter wheel unit (96A357 and MAC6000, Ludl Electronic Products Ltd.). Fluorescence signals were imaged by a scientific complementary metal oxide semiconductor (sCMOS) camera (ORCA flash4.0, Hamamatsu Photonics). The exposure times were 1,500 ms and 500 ms for Sirius and mT-Sapphire, respectively. For monitoring the temperature difference of a HeLa cell, a stable line of HeLa cells ubiquitously expressing gTEMP was incubated at 37°C.

For the IR-laser experiment, HeLa cells stably expressing gTEMP were visualized with the same microscope equipped with dual-view optics (W-VIEW GEMINI, A12801-01, Hamamatsu Photonics) instead of a filter wheel unit for high-speed measurements. A point illumination of the IR-laser (λ = 1,462 nm; FBGLD-1462, SIGMA KOKI) was used for heating cells with a home-made optical system. The IR laser was turned on and off sequentially. Fluorescence signals were imaged by an electron multiplying CCD (EM-CCD) camera (iXon Ultra, Andor Technology). The recording rate was 20 frame per second (fps) with maximum EM-gain in the linear mode. Medium temperature was 28°C.

### Imaging of medaka embryo

Medaka experiment was performed in accordance with the Guidelines for Animal Experimentation of the National Institutes of Natural Sciences, with approval of the Institutional Animal Care and Use Committee of the National Institutes of Natural Sciences. For imaging of medaka embryos, 100 ng/μL mRNA encoding gTEMP was injected into medaka embryos (*Orizias latipes* (OKcab strain)) at the single cell stage according to a standard medaka experimental protocol [16]. The egg envelope (chorion) was removed by hatching enzyme (distributed by the National BioResource Project Medaka) 24–30 hours after the injection. Next, the embryo was put on a 35-mm glass bottom dish and embedded in low-gelling agarose (catalog no. 50081, Lonza Rockland, Inc.) containing 0.03–0.1% artificial sea salt (Rei-sea Marine II; Iwaki Co., Ltd. Tokyo, Japan) to reduce its movement during time-lapse imaging. The embryos were visualized with an inverted microscope (IX-71, Olympus) equipped with a 4× 0.16 numerical aperture dry objective lens (UplanSApo, Olympus), and motorized XY-stage (99A602 Yokogawa Co., Ltd.). Embryos were illuminated using a metal halide lamp (Olympus) through 25% and 6% neutral density filters. The excitation filter FF01-370/36 (Semrock), CFW-Di01-Clin dichroic mirror (Semrock), and emission filters (FF01-447/60 and FF01-520/35 for Sirius and mT-Sapphire, respectively; Semrock) were used with dual-view optics (W-VIEW, A8509-11, Hamamatsu Photonics) to capture two emission channel images simultaneously. Fluorescence signals were imaged by an EM-CCD camera (ImagEM X2-1K, C9100-24B, Hamamatsu Photonics). The images were captured every 5 min for 8–15 hours with a 500 ms exposure time.

### Temperature calibration of gTEMP ratio

Calibration of the ratio of gTEMP and the temperature change were measured and calculated as follows. The temperature of the culture medium was increased by 1–2°C between 34°C and 40°C using a stage top incubator and fluorescence images of gTEMP expressed in HeLa cells were taken at each temperature. The fluorescence intensity ratio of gTEMP changed almost linearly from 30°C to 40°C. Therefore, we estimated the temperature change by extrapolating the fitting curve (linear fitting).

### Data analysis

The relative temperature resolution (*δT*) of gTEMP at each condition was calculated by the following equation,

$$\delta T=\left(\frac{\partial T}{\partial R}\right)\delta R$$

where ∂*T/*∂*R* represents the inverse of the slope of the gTEMP ratio versus temperature diagram and *δR* represents the standard deviation of the averaged gTEMP ratio calculated from the time-lapse images. All images were analyzed by MetaMorph (Molecular Devices, Sunnyvale, CA). The mT-Sapphire/Sirius fluorescence ratio was calculated by dividing the mT-Sapphire intensity by the Sirius intensity within the region of interest. Some figures and movies were made using the ImageJ software.

## Results

### Genetically encoded ratiometric fluorescent thermometer

**Fig 1:**
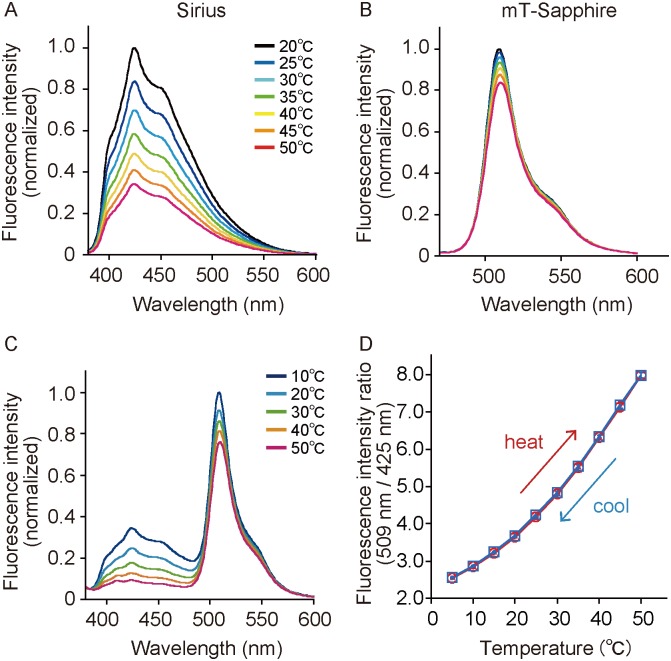
Temperature dependency of Sirius, mT-Sapphire, and equimolar mixtures of Sirius and mT-Sapphire proteins. (A) Temperature-dependent fluorescence spectrum of Sirius. The wavelength at 360 ± 10 nm was used for excitation. (B) Temperature-dependent fluorescence spectrum of mT-Sapphire. The wavelength at 400 ± 10 nm was used for excitation. (C) Temperature-dependent fluorescence spectrum of equimolar mixtures of the two FPs. The wavelength at 360 ± 10 nm was used for excitation. (D) Temperature-dependent fluorescence intensity ratio (open red circle and open blue square) of the equimolar mixtures of the two FPs from 5°C to 50°C. The ratio value was plotted against the solution temperature (*n* = 3). Red and blue lines show the increase in temperature from 5°C to 50°C and the decrease in the opposite direction, respectively. The detailed method for the calculation of the ratio was described in the Methods section. Error bars indicate the standard error (s.e.m.).

Generally, the ratiometric type of fluorescent indicators are more useful for quantitatively measuring a target of interest compared with the intensiometric type because the ratio of the fluorescence intensity is not affected by factors such as the expression level of the indicator itself in cells and the cellular shape. Using a combination of the most and the least temperature sensitive FPs is a simple and effective way to obtain a genetically encoded ratiometric fluorescent thermometer with a high sensitivity. Therefore, we first investigated the temperature-dependent fluorescence intensity and spectra of various FPs. We observed that the fluorescence intensity of Sirius [17], an ultramarine color variant of *Aequoria victoria* GFP, exhibited the largest temperature dependence among the measured FPs, including Sirius, mTurquoise2, mTFP1, EGFP, mT-Sapphire, Venus, TagRFP-T, and mCherry (S1 Fig). Sirius showed a 65% decrease in fluorescence when the environmental temperature was increased from 20°C to 50°C (Fig 1A). In contrast, monomeric T-Sapphire (mT-Sapphire), a variant of GFP [18] that has a mutation (A206K) preventing dimerization, exhibited the smallest temperature dependence with a 17% decrease in fluorescence (Fig 1B). Sirius and mT-Sapphire can be excited with same wavelength (around 360 nm) and their emission wavelengths differ by approximately 85 nm, allowing fast temperature imaging. Therefore, we concluded that the combination of Sirius and mT-Sapphire is ideal as a ratiometric thermometer.

**Fig 2:**
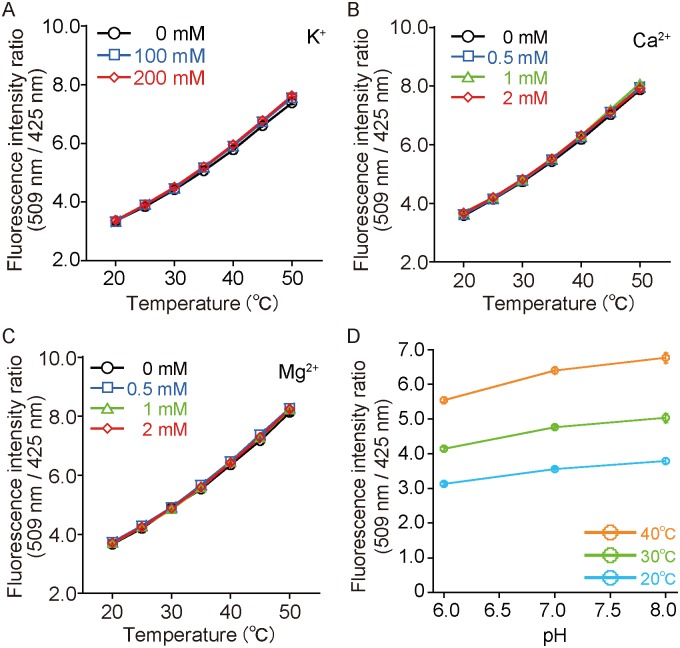
Effects of various ion concentrations on temperature sensing by the equimolar mixtures of Sirius and mT-Sapphire proteins. (A) Temperature-dependent fluorescence intensity ratio of the equimolar mixtures of the two FPs at various K^+^ concentrations. (B) Temperature-dependent fluorescence intensity ratio of the equimolar mixtures of the two FPs at various Ca^2+^ concentrations. (C) Temperature-dependent fluorescence intensity ratio of the equimolar mixtures of the two FPs at various Mg^2+^ concentrations. (D) pH-dependent fluorescence intensity ratio (509/425 nm) of the equimolar mixtures of the two FPs at various temperature. Cyan; 20°C, yellow-green; 30°C, orange; 40°C. A solution containing 30 mM trisodium citrate and 30 mM borax adjusted to pH 6.0, 7.0, and 8.0 was used. Error bars indicate the s.e.m. (*n* = 3).

For a ratiometic measurement of the fluorescence emission, it is necessary that the amount of both FPs should be the same. The fluorescence spectra of purified Sirius and mT-Sapphire proteins mixed in equimolar concentrations showed a clear dependence on the temperature change (Fig 1C). The emission ratio (509 nm/425 nm) increased by 210% when the temperature was changed from 5°C to 50°C (Fig 1D). The ratio change was shown to be 2.6%/°C between 5°C and 50°C (S1 Table). In addition, no hysteresis was observed after a 50°C to 5°C temperature change. Furthermore, the concentrations of K^+^, Mg^2+^, Ca^2+^, and a physiological pH condition did not affect the ratio or the temperature dependency (Fig 2) indicating the usefulness of the system in living cells.

### Monitoring temperature in cells

**Fig 3:**
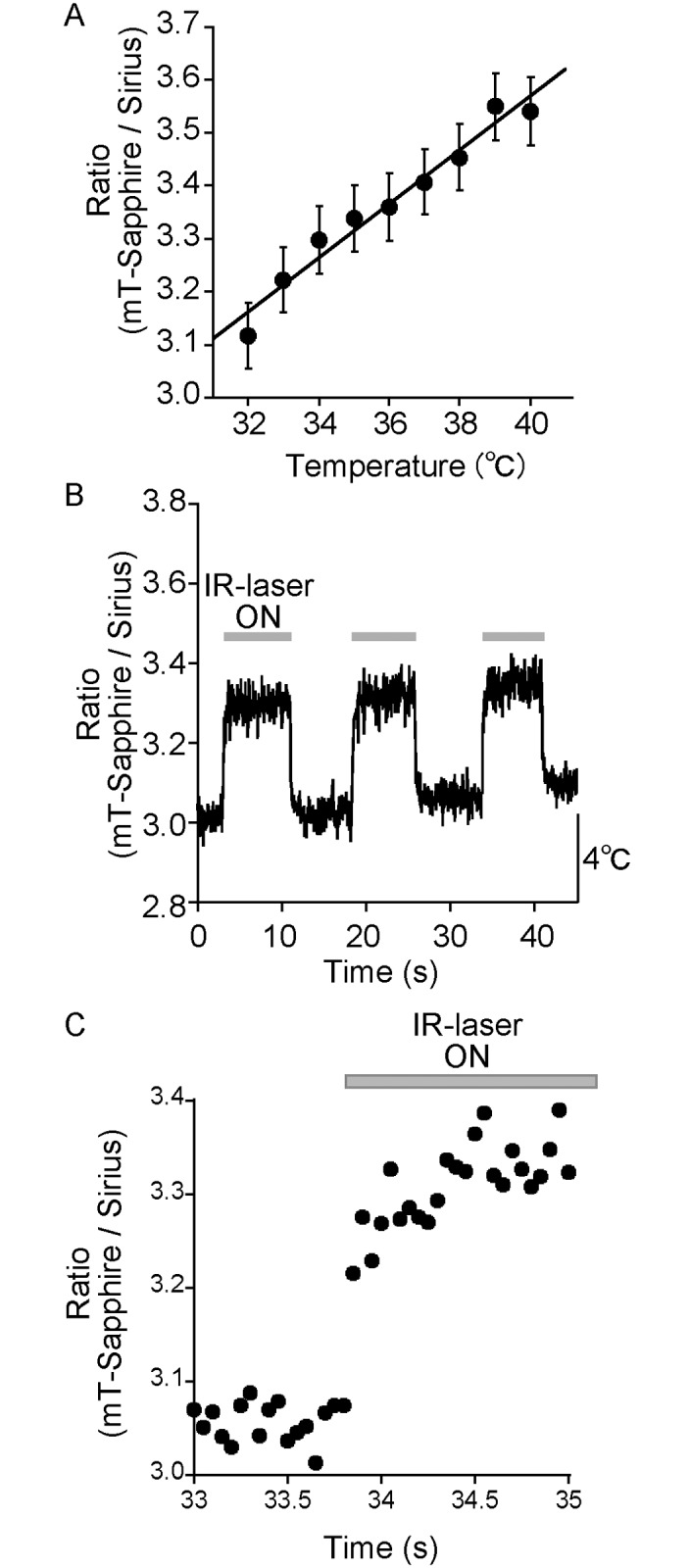
Monitoring temperature in cells with gTEMP. (A) Temperature-dependent fluorescence intensity ratio (black circle) of gTEMP expressed in HeLa cell. The ratio in the cytoplasm was plotted against the cellular medium temperature (*n* = 20). Relative temperature resolution was 0.5°C. (B) Time course of the ratio of gTEMP in a HeLa cell upon IR-laser irradiation. The IR laser was focused in the cytoplasm. We measured and plotted the fluorescence intensity ratio of gTEMP at the focus of the IR laser. The recording rate was 20 fps. The medium temperature was 28°C. (C) The extended figure from (B). Error bars in (A) indicate the s.e.m.

In practice, using the pairing of Sirius and mT-Sapphire as a thermometer would require these FPs to be expressed in a cell with the same stoichiometry. To this end, we first constructed a tandem fusion of Sirius and mT-Sapphire using a linker of two amino acids. However, the ratio change was smaller than that measured using the equimolar mixtures of the FPs (S2 Fig), probably because the Förster resonance energy transfer between the fused proteins deteriorated the indicator performance. Therefore, we independently expressed these FPs and named it as gTEMP (**g**enetically encoded ratiometric fluorescent **TEMP**erature indicator). To express equimolar amounts of Sirius and mT-Sapphire in cells, we linked these FPs with a *Thosea asigna* virus 2A peptide [19]. Using this construction, we succeeded in expressing gTEMP uniformly in the cytoplasm and the nucleus. Next, to test the performance of gTEMP in cells, we measured the fluorescence emission ratio of gTEMP by changing the temperature of the culture medium from 32°C to 40°C. As a result, the ratio of gTEMP increased from 3.1 to 3.5 (Fig 3A), suggesting that gTEMP can behave as a thermometer in cells.

Next, we checked the response speed and reversibility of gTEMP with IR-laser irradiation of a cell because an IR laser can heat water with a high efficiency and cause a temperature increase of the IR-laser irradiated cell [14]. When the IR laser was turned on and off sequentially, we could observe, at 20 fps, a rapid increase and decrease, respectively, of the ratio (Fig 3B and 3C). This result indicates that gTEMP can respond to a rapid temperature change on the order of milliseconds and the heat dissipation occurs on a sub second timescale in cells. Using the calibration curve shown in Fig 3A, 1-mW IR-laser irradiation induced a temperature increase of approximately 5°C. A previous report estimated the temperature increase as 20°C per 7 mW with a focused IR laser (theoretical diameter of the IR-laser spot was approximately 1.4 μm) in a nematode [14]. Therefore, the order of the temperature increase upon IR-laser irradiation was almost consistent with the previous result.

### Monitoring temperature change in mitochondria

**Fig 4:**
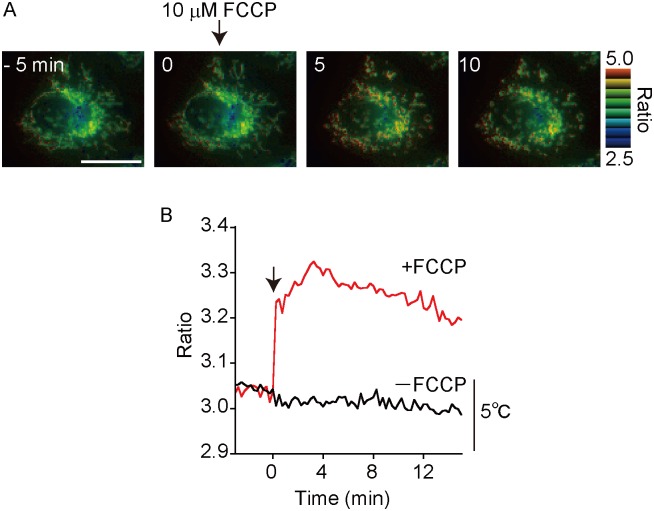
Monitoring temperature change in mitochondria. (A) Pseudo-colored ratio image of gTEMP expressed in mitochondria of a HeLa cell upon FCCP stimulation. At time = 0 min, 10 μM FCCP was added to the cell. (B) Time course of the ratio in mitochondria with FCCP and without FCCP. The temperature scale (5°C) in Fig 4B was estimated from the slope value (0.031 ratio/°C) of S5 Fig. The medium temperature was 37°C. Scale bars indicate 20 μm (A).

Furthermore, gTEMP was expressed in specific intracellular organelles fused with specific localization signal peptides to both FPs (S3 Fig). Since gTEMP is a ratiometric indicator, we can estimate the absolute temperature change in an organelle by calibration with the cell. It is well known that carbonyl cyanide 4-(trifluoromethoxy)phenylhydrazone (FCCP), a proton uncoupling reagent, induces heat production in the mitochondria of living cell [6, 8]. After the addition of 10 μM FCCP to cells expressing gTEMP in mitochondria, we detected a temperature increase (Fig 4 and S4 Fig). The estimated temperature increase at the mitochondria matrix was 6–9°C calculated from the in cell calibration curve as shown in S5 Fig.

### Monitoring temperature distribution in cells

**Fig 5:**
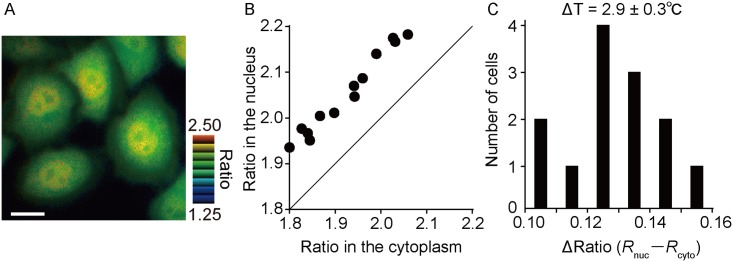
Monitoring temperature difference in cells. (A) Pseudo-colored ratio image of gTEMP ubiquitously expressed in a HeLa cell. (B) Plot of gTEMP ratio in cytoplasm and nucleus regions in each HeLa cell (*n* = 13). (C) Histogram of the gTEMP ratio difference between cytoplasm and nucleus regions converted from (B). The average temperature difference in Fig 5C was 2.9 ± 0.3°C estimated from the slope value (0.045 ratio/°C) of S6 Fig. The medium temperature was 37°C. Scale bar indicates 20 μm (A).

Although the notions of heat production and temperature differences between the cytosol and the nucleus are still controversial [20–23], our measurements using gTEMP suggest that the ratio value in the nucleus region was higher than that in the cytoplasm region under physiological conditions, as previously reported [6, 11] (Fig 5A and 5B). The estimated temperature difference between the cytosol and the nucleus was 2.9 ± 0.3°C (Fig 5C and S6 Fig).

### Monitoring temperature *in vivo*

**Fig 6:**
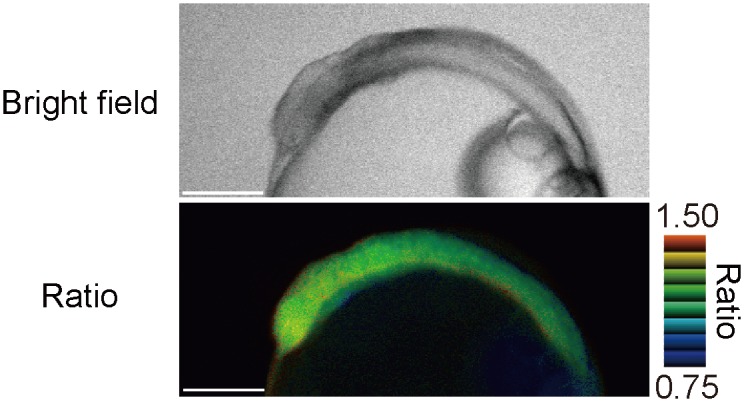
Monitoring temperature in medaka embryo. The bright field image (upper) and pseudo-colored ratio image (bottom) of gTEMP expressed in a medaka embryo. The medium temperature was 25°C. Scale bars indicate 250 μm.

To determine the usability of gTEMP *in vivo*, we injected mRNA of gTEMP into a fertilized egg of medaka fish and successfully observed the fluorescence of gTEMP stably over a long duration (15 hours) without any toxic effect during embryogenesis (Fig 6 and S1 Movie). Therefore, gTEMP has potential as a noninvasive thermometer *in vivo*.

## Discussion

Here, we developed a genetically encoded fluorescence ratiometric thermometer by using a combination of FPs with low sensitivity and high sensitivity to temperature, which were chosen to be mT-Sapphire and Sirius, respectively. The reason why Sirius sensed the temperature change was speculated as follows: Sirius was developed from Y66F variants of GFP [17] and has a lower fluorescence quantum yield than the other FPs we tested (S1 Fig). Thus, the excited state energy in Sirius is easily dissipated through a non-radiative process (i.e., heat generation) to the ground state. Kummer *et al*. indicated that the temperature dependency of the fluorescence lifetime of a Y66F variant of GFP was higher than that of a Y66H variant of GFP [24]. They suggested that hydrogen bonding between a phenolic oxygen atom of the Y66 residue in wild-type GFP and a water molecule plays an important role to fix a π-electron coupling of the chromophore that leads to the emission of photons. However, in the Y66F variant of GFP, the hydrogen bonding is absent because phenylalanine does not possess the phenolic oxygen atom. Therefore, the fixation of the π-electron coupling in the Y66F variant of GFP might be weaker than that of wild-type GFP. Generally, when the temperature is higher, the fluctuation of an atom or molecule is amplified. The fixation of π-electron coupling in the Y66F variant of GFP would be easily affected by the temperature-dependent fluctuation of atoms, indicating the high temperature sensitivity of Sirius.

We showed the usability of gTEMP in intracellular organelles such as mitochondria. As well as some reports on monitoring the temperature increase upon addition of FCCP or CCCP to cells [4, 6, 8, 13], gTEMP succeeded in directly measuring the temperature increase inside mitochondria. One of the physiological parameters, pH, has the potential to affect the gTEMP ratio values. As shown in Fig 2D, gTEMP indicated the pH dependency from pH 6–8, since the p*K*_a_ of mT-Sapphire and Sirius are 4.8 [25] and 3.5 [17], respectively. Thus, we concluded that there is almost no pH sensitivity in gTEMP as long as it is not expressed in an acidic organelle. According to a previous paper by Okabe *et al*., the temperature increase upon FCCP stimulation was reported to be approximately 1°C [6]. However, our result on the temperature increase was 6–9°C. The reason why gTEMP showed such a higher temperature increase is that gTEMP is the first temperature indicator that can be localized and estimate temperature change “inside” mitochondria (mitochondria matrix), whereas other temperature indicators, including fluorescent polymeric thermometer [6], report the temperature “outside” mitochondria. In addition, mitochondria are the venues of heat generation [26]. Therefore, the value of the temperature changes between gTEMP and other indicators, except for tsGFPs [8], should be different.

Although a heterogeneous temperature distribution in a single cell is still controversial [20–23], our results have also showed the temperature difference between the nucleus and cytoplasm in a cell and supports the existence of heterogeneous temperature distribution, as has been previously reported [6, 11, 13]. Although we could not clearly determine why the temperature in the nucleus is higher than cytoplasm, we speculated on the possible mechanisms for the temperature difference as follows: (1) the nuclear envelope blocks heat diffusion from the nucleus to cytosol; (2) the speed of heat diffusion in the nucleus could be different in the cytosol; (3) the continuous production of strong heat may occur in the nucleus. If (3) is true, the energy used for nuclear heat production may not be consistent with the total cellular energy consumption. Further study should be performed to clarify this.

Kiyonaka *et al*. suggested that an organelle-targetable thermometer is essential for monitoring the temperature at intracellular organelles because the generated heat is diffused immediately [21]. Actually, each gTEMP and tsGFP [8] localized at the mitochondria matrix, which is the heat source after addition of a proton uncoupler, and monitored the temperature increase. Therefore, our data bring us close to Kiyonaka’s vision.

Furthermore, gTEMP has a wide range of temperature sensitivity from 5°C to 50°C and an ability to track fast temperature change on the order of milliseconds. Therefore, gTEMP can be widely used for not only *in vivo* monitoring of various living species but also heat generation and temperature difference at local areas such as mitochondria and nucleus.

## Accession code

GenBank/EMBL/DDBJ: the nucleotide sequence of gTEMP has been submitted under the Entry ID: LC132716.

## Supporting information

S1 Fig Temperature-dependent relative fluorescence intensity of various FPs. Relative fluorescence intensities were calculated by dividing each peak value of the fluorescence spectrum by the peak value at 20°C.
(TIF)

S2 Fig Difference of temperature-dependent fluorescence intensity ratio between the equimolar mixtures of the two FPs and fusion FPs. The ratio of the equimolar mixtures of the two FPs (black line) and Sirius-mT-Sapphire fusion construction (grey line). Error bars indicate the s.e.m. (*n* = 3).
(TIF)

S3 Fig Organelle-specific temperature sensing. gTEMP can localize in mitochondria (A) and nuclei (B) of HeLa cells using each localization signal. Left panels: Sirius channel, middle panels: mT-Sapphire channel, right panels: Ratio. The exposure times of the sCMOS camera were 900 ms and 300 ms for Sirius and mT-Sapphire, respectively. The medium temperature was 37°C. Scale bars indicate 20 μm.
(TIF)

S4 Fig Influence of FCCP on gTEMP. (A) Fluorescence spectra of gTEMP purified protein in the presence and absence of 10 μM FCCP. The measurement was performed at 37°C. Error bars indicate the s.e.m (*n* = 3). (B) Fluorescence intensity ratio (509/405 nm) calculated from (A). Error bars represent the s.e.m. Addition of 10 μM FCCP increased the gTEMP ratio by 3.5%.
(TIF)

S5 Fig Calibration curve of the gTEMP ratio for mitochondria. Temperature-dependent ratio of gTEMP expressed in mitochondria of a HeLa cell with the same conditions as those used for Fig 4. The average ratio (black circle) was plotted against the medium temperature. The temperature increase upon FCCP stimulation of Fig 4B was estimated from the slope value (0.031 ratio/°C). Error bars indicate the s.e.m. (*n* = 10). Relative temperature resolution was 0.4°C.
(TIF)

S6 Fig Calibration curve of the gTEMP ratio for cytoplasm. Temperature-dependent ratio of gTEMP stably expressing in a HeLa cell with the same measurement conditions as those used for Fig 5. The average ratio (black circle) was plotted against the medium temperature. The temperature difference between the cytoplasm and nucleus of Fig 5C was estimated from the slope value (0.045 ratio/°C). Error bars indicate the s.e.m. (*n* = 13). Relative temperature resolution was 0.1°C.
(TIF)

S1 Table Comparison of the properties among fluorescent indicators for intracellular temperature. Signal change was calculated for each indicator according to a previously described protocol^1^.
(DOCX)

S2 Table Oligonucleotides used in this study. (DOCX)

S1 Movie Time-lapse movie of the pseudo-colored gTEMP ratio of medaka embryo. We monitored the temperature distribution during embryogenesis for 15 hours *in vivo*. Images were acquired every 5 minutes. The medium temperature was 25°C. Scale bar indicates 250 μm.
(AVI)
