# Supplementary figures and images for: Genetically encoded ratiometric fluorescent thermometer with wide range and rapid response
Source: PLoS One. 2017 Feb 17;12(2):e0172344. doi: 10.1371/journal.pone.0172344 (PMC5315395; doi:10.1371/journal.pone.0172344)

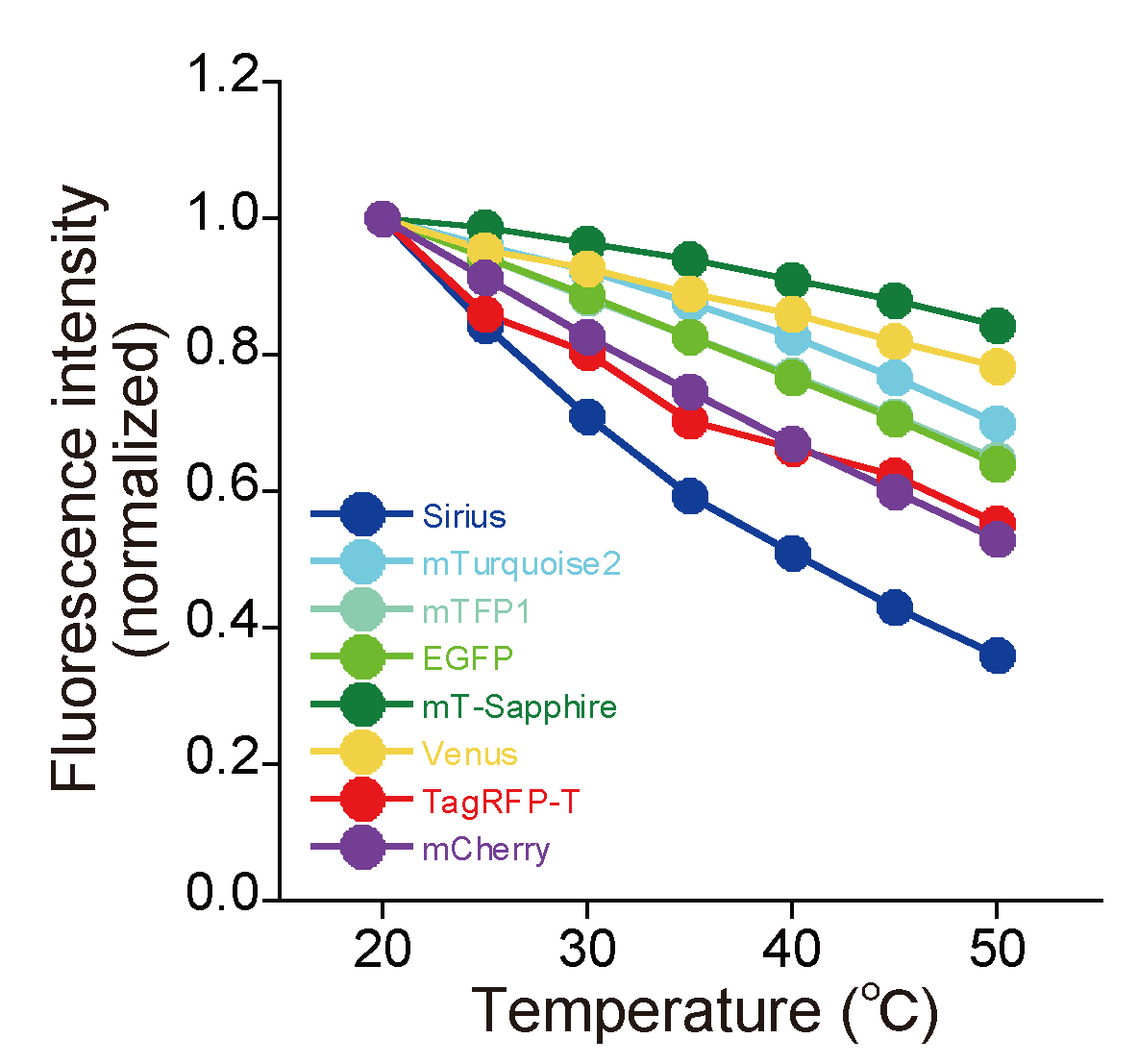

Supplement: S1 Fig — Relative fluorescence intensities were calculated by dividing each peak value of the fluorescence spectrum by the peak value at 20°C. (TIF) [file pone.0172344.s001.tif]

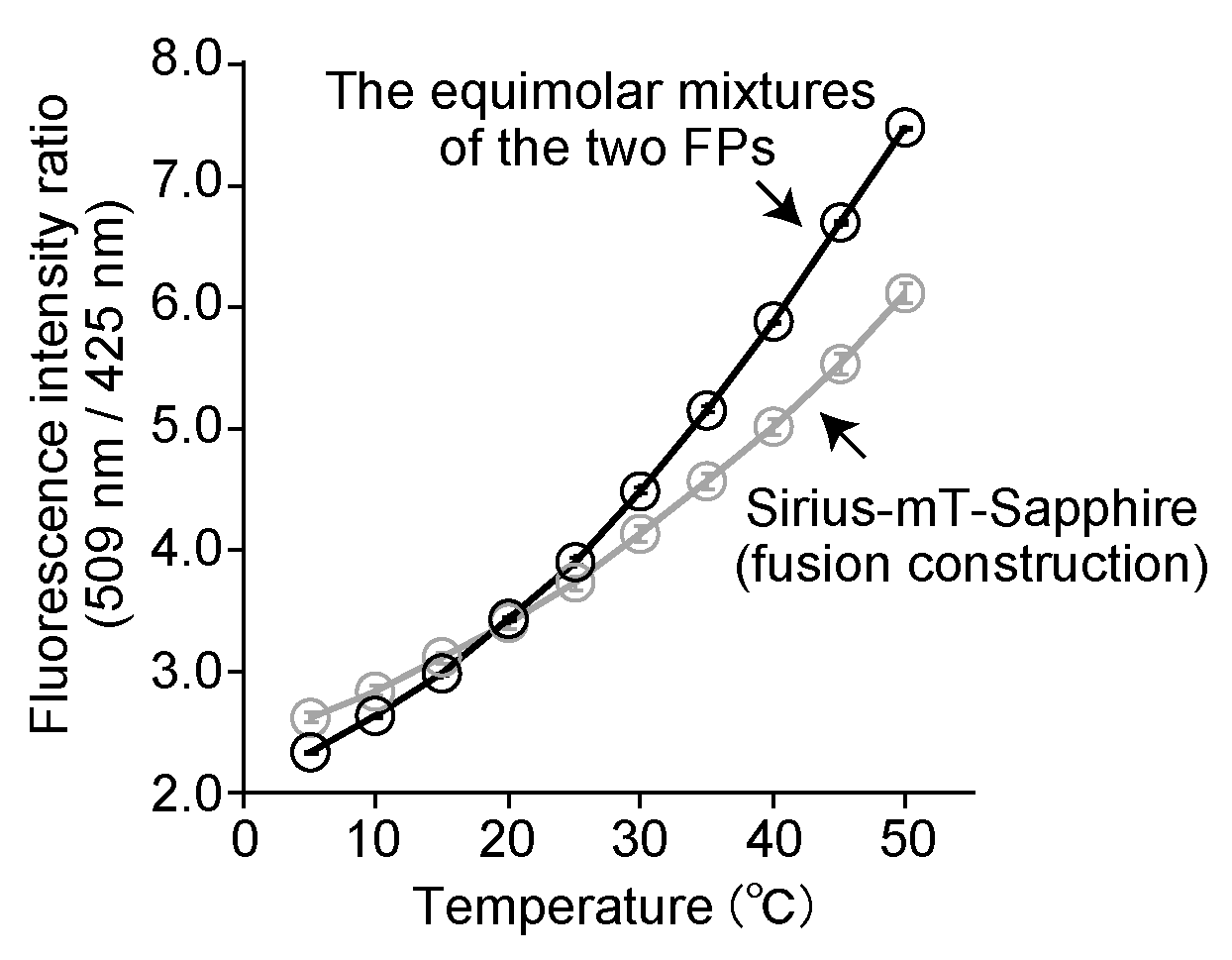

Supplement: S2 Fig — The ratio of the equimolar mixtures of the two FPs (black line) and Sirius-mT-Sapphire fusion construction (grey line). Error bars indicate the s.e.m. (n = 3). (TIF) [file pone.0172344.s002.tif]

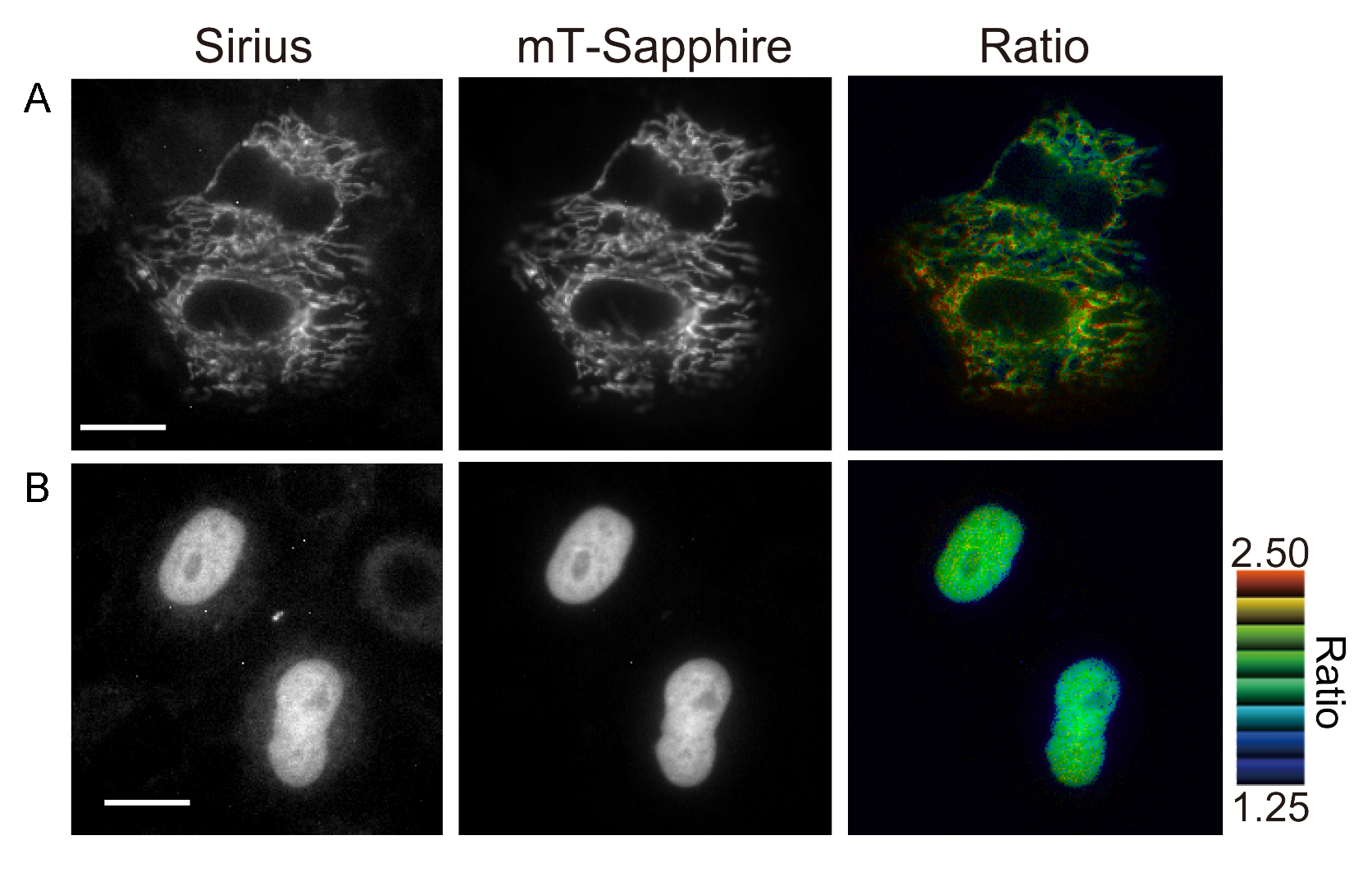

Supplement: S3 Fig — gTEMP can localize in mitochondria (A) and nuclei (B) of HeLa cells using each localization signal. Left panels: Sirius channel, middle panels: mT-Sapphire channel, right panels: Ratio. The exposure times of the sCMOS camera were 900 ms and 300 ms for Sirius and mT-Sapphire, respectively. The medium temperature was 37°C. Scale bars indicate 20 μm. (TIF) [file pone.0172344.s003.tif]

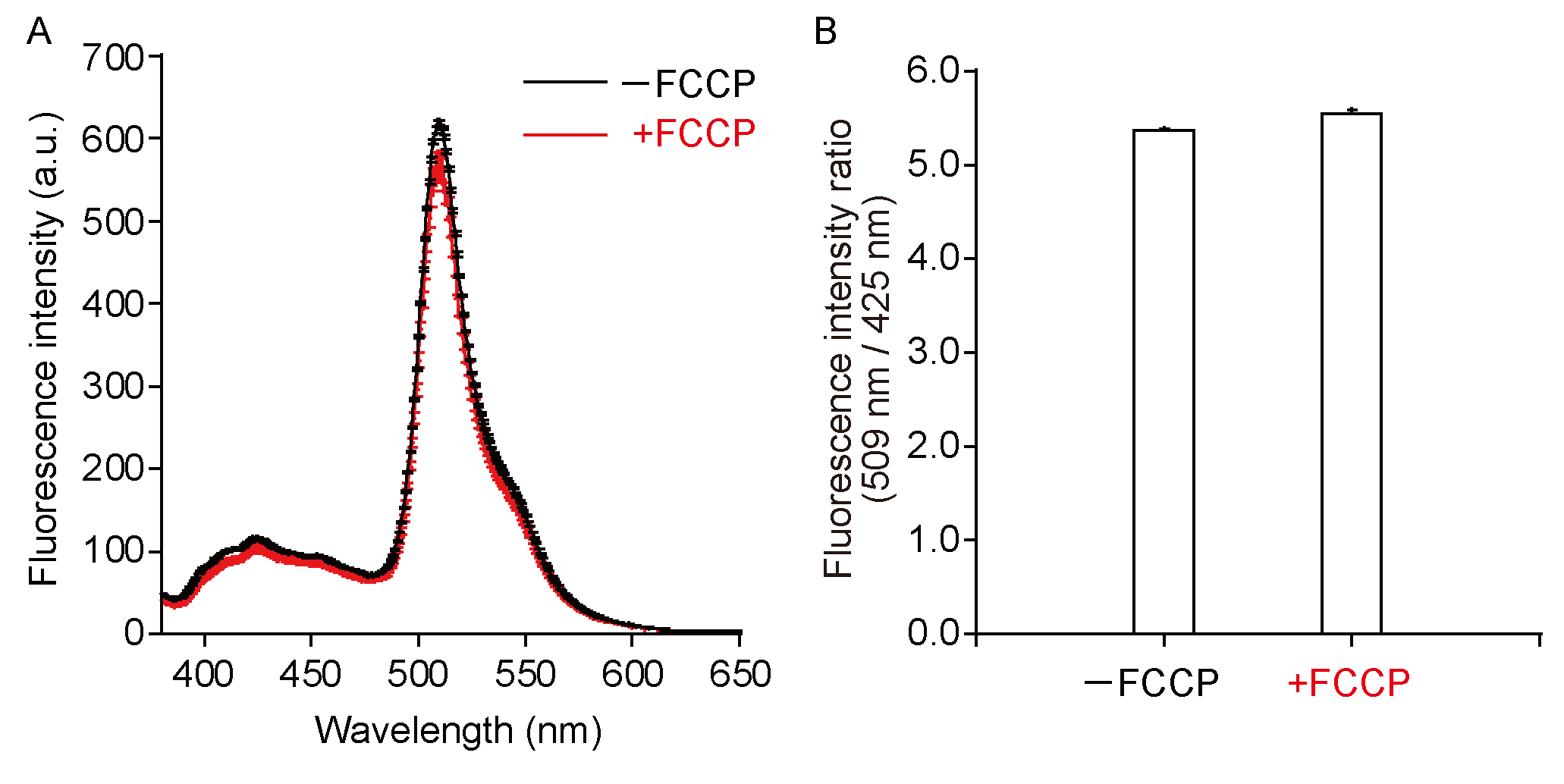

Supplement: S4 Fig — (A) Fluorescence spectra of gTEMP purified protein in the presence and absence of 10 μM FCCP. The measurement was performed at 37°C. Error bars indicate the s.e.m (n = 3). (B) Fluorescence intensity ratio (509/405 nm) calculated from (A). Error bars represent the s.e.m. Addition of 10 μM FCCP increased the gTEMP ratio by 3.5%. (TIF) [file pone.0172344.s004.tif]

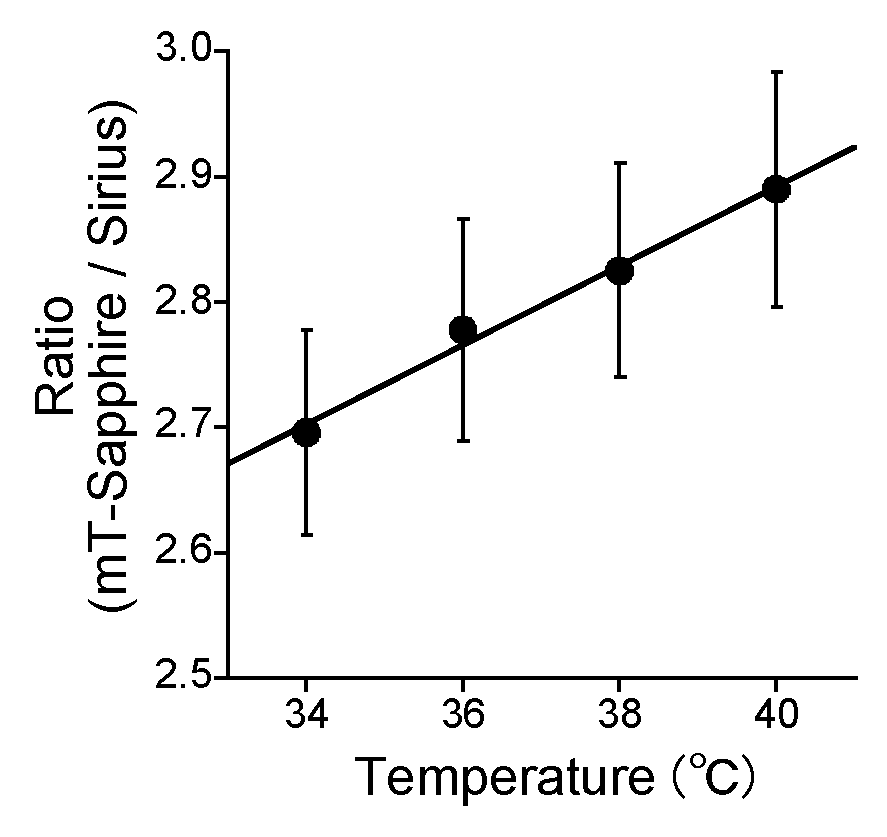

Supplement: S5 Fig — Temperature-dependent ratio of gTEMP expressed in mitochondria of a HeLa cell with the same conditions as those used for Fig 4. The average ratio (black circle) was plotted against the medium temperature. The temperature increase upon FCCP stimulation of Fig 4B was estimated from the slope value (0.031 ratio/°C). Error bars indicate the s.e.m. (n = 10). Relative temperature resolution was 0.4°C. (TIF) [file pone.0172344.s005.tif]

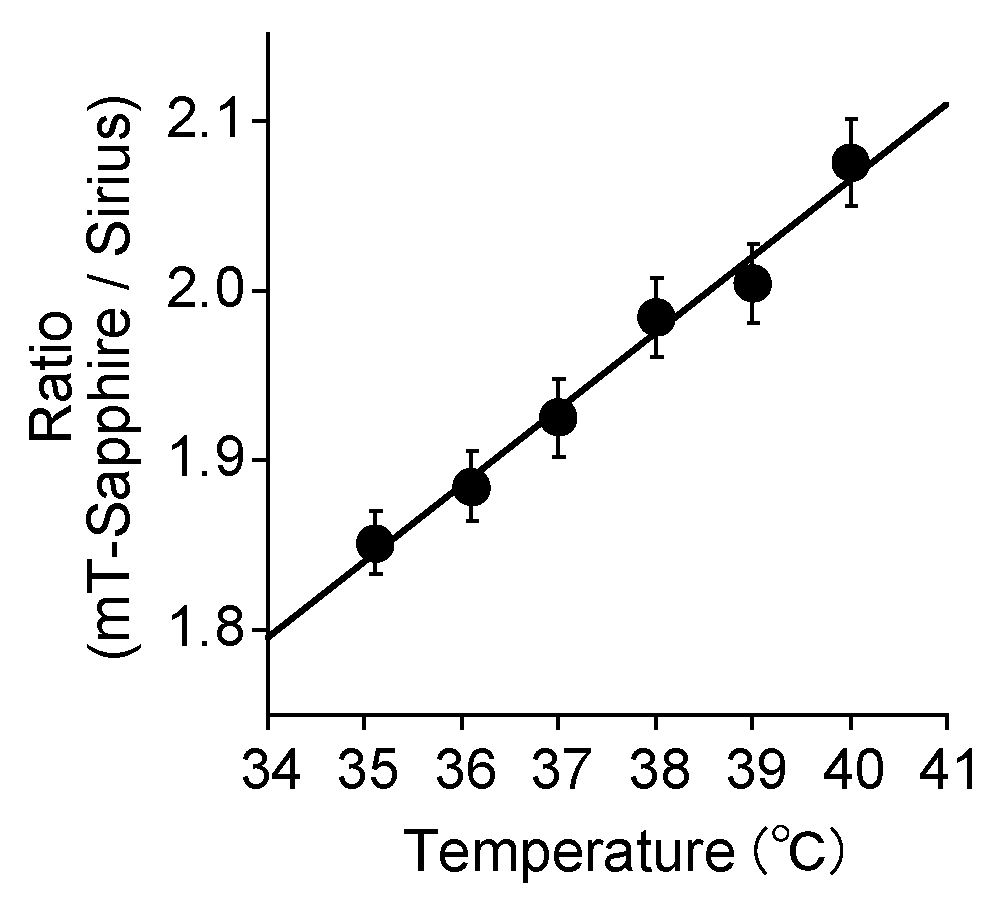

Supplement: S6 Fig — Temperature-dependent ratio of gTEMP stably expressing in a HeLa cell with the same measurement conditions as those used for Fig 5. The average ratio (black circle) was plotted against the medium temperature. The temperature difference between the cytoplasm and nucleus of Fig 5C was estimated from the slope value (0.045 ratio/°C). Error bars indicate the s.e.m. (n = 13). Relative temperature resolution was 0.1°C. (TIF) [file pone.0172344.s006.tif]
