# Supplementary material for: Genetically encoded ratiometric fluorescent thermometer with wide range and rapid response
Source: PLoS One. 2017 Feb 17;12(2):e0172344. doi: 10.1371/journal.pone.0172344 (PMC5315395; doi:10.1371/journal.pone.0172344)
Supplement: S1 Table — Signal change was calculated for each indicator according to a previously described protocol1. (DOCX) [file pone.0172344.s007.docx]

|  | Temperature range  (°C) | Signal change  (%/°C) | Number of excitation wavelength | Number of emission wavelength | *in vivo* applicable? |
| --- | --- | --- | --- | --- | --- |
| gTEMP | 5 – 50 | 2.6 | 1 | 2 | Yes |
| tsGFP1^2^ | 35 – 40 | 3.0 | 2 | 1 | Yes |
| tsGFP2^2^ | 40 – 45 | 3.1 | 2 | 1 | Yes |
| EGFP^3^ | 20 – 65 | 2.1 | 1 | 1 | Yes |
| FPT^4^ | 20 – 50 (30 – 40) | 4.8 (6.3) | 1 | 1 | difficult |

References

S1. Carlos D. S. Brites *et al.* *Nanoscale,* **4**, 4799-4829 (2012).

S2. Kiyonaka, S. *et al.* *Nat. Methods*, **10**, 1232-1238 (2013).

S3. Kamei, Y. *et al.* *Nat. Methods*, **6**, 79– 81 (2009).

S4. Okabe, K. *et al.* *Nat. Communications* **3**, Article number: 705 (2012).
