# Supplementary material for: Genetically encoded ratiometric fluorescent thermometer with wide range and rapid response
Source: PLoS One. 2017 Feb 17;12(2):e0172344. doi: 10.1371/journal.pone.0172344 (PMC5315395; doi:10.1371/journal.pone.0172344)
Supplement: S2 Table — (DOCX) [file pone.0172344.s008.docx]

| Primer | Oligonucleotide sequence (5’ to 3’) |
| --- | --- |
| T-Sapphire-A206K-F | TACCTGAGCATCCAGTCCAAGCTGAGCAAAGACCCCAAC |
| pRSET_B_-*BamH*I-FP-F | AGCTCGGATCCCATGGTGAGCAAGGGCGAG |
| FP-stop-*EcoR*I-R | AGCTTCGAATTCTTACTTGTACAGCTCGTCCATGC |
| *Xho*I-FP-F | AATTCTCGAGATGGTGAGCAAGGGCGAGGAG |
| *Xho*I-Δstop-FP-R | CTCAGCTCGAGCTTGTACAGCTCGTCC |
| pcDNA3-*BamH*I-FP-F | AGCTCGGATCCCACCATGGTGAGCAAGGGCGAG |
| *Sph*I-Δstop-FP-R | GCCATGCATGCGCTTGTACAGCTCGTCCATG |
| *Sph*I-T2A-*Sac*I-F1 | CATGGCAGCGGCGAGGGCAGAGGCAGCCTGCTGACCTGC |
| *Sph*I-T2A-*Sac*I-F2 | GGCGACGTGGAGGAGAACCCCGGCCCCGAGCT |
| *Sph*I-T2A-*Sac*I-R1 | TCAGCAGGCTGCCTCTGCCCTCGCCGCTGCCATGCATG |
| *Sph*I-T2A-*Sac*I-R2 | CGGGGCCGGGGTTCTCCTCCACGTCGCCGCAGG |
| *Sac*I-FP-F | GATAAGAGCTCATGGTGAGCAAGGGCGAGG |
| *BamH*I-coxVIII-F | AGCTCGGATCCGCCACCATGTCTGTTCTGACTC |
| *Not*I-coxVIII-R | CATGATGCGGCCGCGCTTGGGATCGC |
| *Not*I-FP-F | CAAGCGCGGCCGCATCATGGTGAGCAAGGGCGAGG |
| *Sac*I-coxVIII-F | GATAAGAGCTCATGTCTGTTCTGACTCCTCTGC |
| pcDNA3-*Hind*III-FP-F | GGATCAAGCTTGCCACCATGGTGAGCAAGGGCGA |
| *EcoR*I-Δstop-FP-R | CGATCGAATTCCTTGTACAGCTCGTCCATGCTC |
| *Sph*I-histone2B-R | GCCATGCATGCGCTTAGCGCTGGTGTAC |
| *Xho*I-histone2B-stop-R | CTCAGCTCGAGTTACTTAGCGCTGGTGTAC |

FP: Fluorescent protein (Sirius or mT-Sapphire)
